# Dynamic modulation of DC-SIGN and FcΥR2A receptors expression on platelets in dengue

**DOI:** 10.1371/journal.pone.0206346

**Published:** 2018-11-09

**Authors:** Sojit Tomo, Sindhujadevi Mohan, Vijaykumar Shettikothanuru Ramachandrappa, Daisy Mariya Samadanam, Sevanthy Suresh, Agieshkumar Balakrishna Pillai, Kadhiravan Tamilarasu, Rameshkumar Ramachandran, Soundravally Rajendiran

**Affiliations:** 1 Department of Biochemistry, Jawaharlal Institute of Post-graduate Medical Education and Research (JIPMER), Puducherry, India; 2 CIDRF, Puducherry, India; 3 Department of Medicine, Jawaharlal Institute of Post-graduate Medical Education and Research (JIPMER), Puducherry, India; 4 Department of Paediatrics, Jawaharlal Institute of Post-graduate Medical Education and Research (JIPMER), Puducherry, India; University of Malaya, MALAYSIA

## Abstract

Platelet activation has been reported to play a major role in inflammatory response and thrombocytopenia during dengue viral infection. Cells expressing FcϒR2Aand DC-SIGN receptors are reported to be involved in dengue virulence. The present study is designed to assess the expression level of these two receptors on platelet surface collected from dengue patients and to study its association in patients with platelet RNA positive for dengue virus. This was an analytical cross-sectional study carried out in JIPMER hospital, Puducherry. Forty-four patients with dengue infection as cases and 44 patients with non dengue acute other febrile illness(OFI) as controls were recruited. Peripheral venous blood was withdrawn on day of admission, day 3 post admission and day of discharge and serological tests for NS1 dengue antigen and anti IgM antibody were analyzed for diagnosis of dengue infection. Platelet rich plasma was assessed for DC SIGN, FcϒR2A levels and platelets separated from dengue patients were subjected to RNA extraction and detection of presence of viral RNA. The study observed a decreased expression of DC-SIGN and FcϒR2A on platelets in dengue patients compared to OFI group on all the time points. Further, cells expressing DC-SIGN and FcϒR2A were found to be decreased on platelets in dengue patients who were positive for NS1 antigen. DC-SIGN and FcϒR2A expression was also found to be notably decreased in patients positive for platelet DENV RNA when compared with patients negative for platelet DENV RNA. Our results suggest that DC-SIGN and FcϒR2A, which are receptors for viral capture and immune mediated clearance respectively, might be down regulated on platelets in patients with dengue infection. The decreased receptor expression diminishes platelet activation and subsequently has protective action on the host from the ongoing conflict between immune system and dengue virus.

## Introduction

Dengue viral infection causes self-limiting mild dengue fever to life threatening severe dengue. The actual mechanism that regulates the disease virulence is not completely understood. Some of the earlier studies by several groups, including ours, have documented involvement of oxidative stress and macrophage activation markers in dengue severity.[1] Though thrombocytopenia is a common feature in dengue syndrome, the roles played by platelets in the platelet pathobiology remains poorly deciphered mechanism. Interestingly a very recent study has reported an altered platelet proteome in dengue that may open new pathways to understand the disease pathogenesis. [2]

Dengue is the most prevalent human arbovirus disease worldwide. Dengue virus (DENV) infection causes syndromes varying from self-limiting febrile illness to severe dengue. Although dengue pathophysiology is not completely understood, it is widely accepted that increased inflammation plays important roles in dengue pathogenesis. Platelets are blood cells, classically known as effectors of hemostasis, which have been increasingly recognized to have major immune and inflammatory activities. Nevertheless, the phenotype and effector roles of platelets in dengue pathogenesis are not completely understood.

DC-SIGN,(Dendritic Cell-Specific Intercellular adhesion molecule-3-Grabbing Non-integrin) also known as CD209, is a membrane receptor which has been found to play a role in dengue infection of dendritic cells and macrophages. DC-SIGN acts as a receptor for the high mannose N-linked glycans moieties present on viral envelope. This helps the virus to adhere to the target cells. DC-SIGN also supports primary immune response by stabilizing the engagement of T cell receptor with dendritic cells.[3] Dengue virus lacking glycosylated residues demonstrated reduced infectivity of cells.[4] Reciprocally, cells expressing higher levels of DC-SIGN are more susceptible to dengue infection.[5,6] Hence, DC-SIGN has a vital role in mediating dengue viral infection of cells. Recently, platelets also, as demonstrated by Chaipan C et al and Hottz ED et al, have been shown to express DC-SIGN on its surface.[7,8]

FCϒR2A, also known as CD32, is the major Fc receptor present on platelets. They form the crucial link in the immune-mediated destruction of platelets. Antibodies produced against dengue virus affect platelets in manifold ways. FcƳR2A, which binds with Immunoglobulins produced against dengue virus, makes the platelet vulnerable to phagocytosis by macrophages. Beltrán D et al shows that NK cells can also identify and destroy antibody-coated platelets by ADCC.[9] In addition, FcƳR2A also plays a dominant role in the antibody-dependent enhancement of viral infection, leading to a more severe disease spectrum. In order to understand the role of above mentioned receptors on surface of platelets in dengue patients, we evaluated the expression of DC-SIGN and FcƳR2A on platelets in dengue and other febrile illness throughout the course of infection and its association in patients with platelet RNA positive for dengue virus.

## Materials and methods

This was an analytical cross-sectional study carried out in collaboration between Department of Biochemistry, Medicine and Pediatrics of Jawaharlal Institute of postgraduate medical education and research (JIPMER), Puducherry during the period from January 2016 and November 2017.

The study subjects were adults and children who came to Jawaharlal Institute of postgraduate medical education and research (JIPMER), Puducherry with febrile illness. Forty Four patients with dengue infection and Forty Four patients with non dengue acute febrile illness were recruited as cases and controls respectively. Three ml of peripheral venous blood was withdrawn from children and 5 ml from adults at day of admission, day 3 and day of discharge. Serological tests for NS1 dengue antigen and anti IgM antibody were analyzed for diagnosis of dengue infection by ELISA. Complete blood count and routine biochemical parameters were obtained from hospital information system.

### Ethics statement

The study was approved by the Institute Ethics Committee (Human Studies), JIPMER (Ref no. JIP/IEC/2015/21/733). Written informed consent was obtained from all adult subjects and from parent or guardian of child participants. All procedures followed were done in accordance with the Helsinki Declaration of 1975, as revised in 2013.

### Flow cytometric analysis

Platelets were separated from Acid Citrate Dextrose anti-coagulated venous blood by centrifugation method. FACS Calibur 4-colour cytometer (Becton Dickinson, California, USA) was used to analyze the isolated Platelets based on their immunofluorescence. Surface markers on Platelets were analyzed using the antibodies conjugated to phycoerythrin (PE), peridinin chlorophyll protein (PerCP) and BB515. Antibodies used were PE Mouse Anti-Human CD32 Clone FLI8.26, BB515 Mouse Anti-Human CD209 Clone DCN46 and PerCP-Cy^Tm^5.5 Mouse Anti-Human CD41 Clone HIP8. Ten thousand Platelets were collected for analyses of each fluorochrome-conjugated surface marker. CD41 (Platelet marker), CD209 (DC-SIGN), CD32 (FCϒR2A) were analyzed as per the BD Pharmingen’s (BD Biosciences, California, USA) technical protocol.

### Platelet isolation

Separation of Platelets Rich Plasma (PRP) from ACD anticoagulated blood (1:9) was carried out by centrifugation method. Whole blood, after allowing to settle for 30 min, was subjected to centrifugation at 300 rpm for 25 min at room temperature. The upper PRP layer was pipetted out into a fresh 2mL centrifuge tube and was again subjected to centrifugation at 900g for 10 min at room temperature. Platelet pellet was formed at the bottom of the tube. The supernatant (Platelet Poor Plasma) was discarded and 1mL Tyrode’s buffer was added for washing the platelet pellet. Centrifugation was done at 800g for 5 min at room temperature. The supernatant was discarded and the platelet pellet was stored in 1mL Tyrode’s buffer at -80°C.

### RNA extraction

RNA extraction from isolated platelets was done by the Trizol method. After preparing RNase free environment, 400μL of isolated platelets was added to 800μL of Trizol reagent and was mixed well with the vortex. 300μL of Chloroform was added for phase separation and was subjected to centrifugation at 12000 rpm for 15 min at 4°C. The aqueous layer formed is separated without disturbing the lower organic layer. 500 μL of Isopropanol was added to the aqueous layer to precipitate nucleic acids. Centrifugation was done at 12000 rpm for 10 min at 4°C leading to pellet formation. The supernatant was discarded without disturbing the pellet. 1mL of 75% Ethanol was added for washing and was again subjected to centrifugation at 12000 rpm for 10 min at 4°C. After discarding the supernatant, the pellet was dried in laminar air flow. The pellet obtained was resuspended in 30 μL of Nuclease-Free Water and was heated in the dry bath at 60°C. RNA extracted was assessed using Nanodrop Spectrophotometer and was stored at -80°C.

### cDNA conversion

cDNA conversion of RNA template was done using RT Master mix (Applied Biosystems). Reaction Master Mix was prepared using 10X RT Buffer (2 μL), 25X dNTP Mix 100nM (0.8 μL), 10X RT Random primers (2 μL), MultiScribe^TM^ Reverse Transcriptase (1 μL), RNAase Inhibitor (1 μL) and Nuclease-Free Water (3.2 μL). 1000ng of RNA template was taken. The reaction volume was made up to 20μL. Thermal cycler conditions were set for activation of enzyme at 25°C for 10 min, cDNA synthesis 37°C for 120 min and inactivation of enzyme 25°C for 10 min followed by Holding at 4°C. The cDNA obtained was stored at 4°C.

### Real-Time PCR

Real-Time PCR was done using SYBR Green Master Mix. Reaction Mixture was prepared using 2X SYBR Master Mix (10 μL), Dengue Universal Forward Primer 10μM (0.4 μL) (TTGAGTAAACYRTGCTGCCTGTAGCTC), Dengue Universal Reverse Primer 10μM (0.4μL) (GAGACAGCAGGATCTCTGGTCTYTC) and Nuclease-Free Water (7.2 μL). The primers were obtained from Integrated DNA Technologies (IDT). The reaction volume was made up to 20μL by adding 2 μL cDNA template. Thermal profile conditions were set for initial denaturation at 95°C for 30sec, denaturation at 95°C for 5sec & Annealing at 60°C for 30 sec for 40 cycles followed by melt curve. The reaction was done in duplicates with a No Template Control. Good Amplification and adequate fluorescence were obtained.

### Statistical analysis

All categorical and qualitative variables were presented as frequencies and percentages. Normality of the continuous data was tested by Kolmogorov–Smirnov test and accordingly appropriate parametric or non-parametric tests were used. Non-Gaussian data was presented as median with inter-quartile range. Mann-Whitney U test was used for the comparison of hematological and biochemical parameters between cases and controls as well as the comparison of DC-SIGN and FCϒR2A expression in different dengue subgroups. Kruskal Wallis Rank test with Dunn’s post-test was used to compare DC-SIGN and FCϒR2A expression across Dengue, Other febrile illness group and Non febrile group. Friedman test with Dunn’s post-test was used to compare DC-SIGN and FCϒR2A expression between DOA, Day3 and DOD. All statistical analysis was carried out at 95% confidence interval and p value <0.05 considered statistically significant. All statistical analysis was carried out using SPSS version 19 (Armonk, NY: IBM Corp).

## Results

Forty-Four patients with dengue and Forty-Four patients with OFI were recruited in the study. Among the dengue cases, 35 were adults and 9 were pediatric patients. Within the OFI cases, 36 were adults and 8 were pediatric patients. Out of 44 cases of Dengue, 34 had non severe dengue and 10 had severe dengue. Platelet count and WBC count was significantly decreased (p = 0.018; p = 0.006) and AST was significantly increased (p = 0.002) in Dengue patients when compared with OFI. (Table 1)

**Table 1:** Comparison of hematological and biochemical parameters in dengue and other febrile illness during admission.

Parameters	Dengue Median (IQR) N = 44	OFI Median (IQR) N = 44	P value
PlateletCount (10^3^/cu.mm)	61 (33.5–153.5)	129.5 (67–234.5)	0.018*
Hemoglobin (g/dl)	12.9 (10.8–14.4)	12.6 (10–14.2)	0.556
HCT(%)	37.15±10.8 (32.05–42.85)	37.5 (31.45–42.25)	0.897
MCV(fL)	82.6 (77.2–86.1)	84.85 (81.55–88.95)	0.084
MCH(pg)	28.4 (26–29.4)	28.45 (26.8–29.35)	0.759
MCHC(gm%)	33.9 (32.3–34.9)	33.3 (32.6–33.9)	0.084
WBC (10^3^/cu.mm)	5.01 (4.29–7.36)	8.21 (4.39–11.67)	0.006*
Monoctye(%)	2.3 (1.8–5)	2.1 (1.4–3.7)	0.072
Lymphocyte(%)	38.75 (25.55–47.95)	31.2 (14.6–53.6)	0.234
Urea (mg/dl)	20 (14–28)	19 (15–32)	0.693
Creatinine (mg/dl)	0.85 (0.73–0.95)	0.83 (0.7–0.95)	0.603
AST (IU/L)	95 (48–275)	51 (27–99)	0.002*
ALT (IU/L)	68 (28–132)	39 (22–115)	0.105

Statistical Test: Wilcoxon rank sum (Mann Whitney) test

*p value <0.05 –statistical significance

### DC-SIGN and FCϒR2A expression on platelets in dengue-infected patients when compared with other febrile illness

We subjected the platelets on Day of admission from the three study groups (Dengue patients, Other Febrile illness, and Non Febrile illness) for flow cytometry analysis.

**Fig 1:**
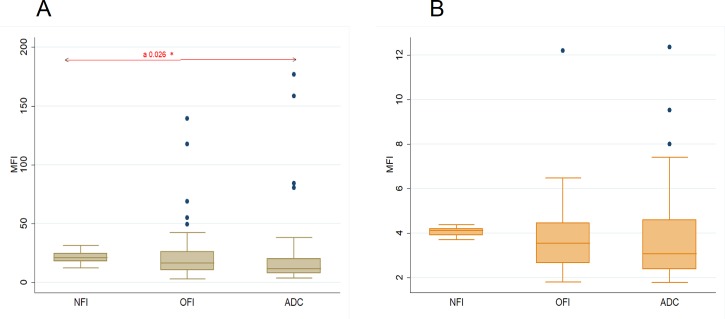
**Comparison of MFI among different groups on day of admission (A) DC-SIGN (CD209) (B) FCϒR2A (CD32).** (DOA–Day of Admission, Dengue—All Dengue Cases, NFI- Non Febrile Illness, OFI- Other Febrile Illness) *p value <0.05 –statistical significance Statistical Test: Kruskal Wallis Rank test a- Indicates statistical significance between all groups on day of admission.

Mean fluorescence intensity (MFI) of DC-SIGN was found to be decreased in Dengue Patients when compared with Other Febrile Illness and Non Febrile Illness. The decline in DC-SIGN expression was found to be statistically significant (p = 0.026) on comparison across all groups. On post hoc comparison, a statistically significant difference in DC-SIGN expression was seen between Dengue and Nonfebrile illness group.(Fig 1A) Mean fluorescence intensity (MFI) of FCϒR2A was also found to be decreased in Dengue Patients when compared with Other Febrile Illness and Non febrile Illness, though not statistically significant.(Fig 1B)

To gain information on the pattern of expression of DC-SIGN and FCϒR2A along with the course of the disease, we analyzed the platelets from Day 3 and DOD using flow cytometry. We found that both DC-SIGN and FCϒR2A expression increased from DOA to Day 3 and to DOD in Dengue cases. Statistically significant difference was seen only in FCϒR2A expression (p = 0.0001) on comparison across all groups.

**Fig 2:**
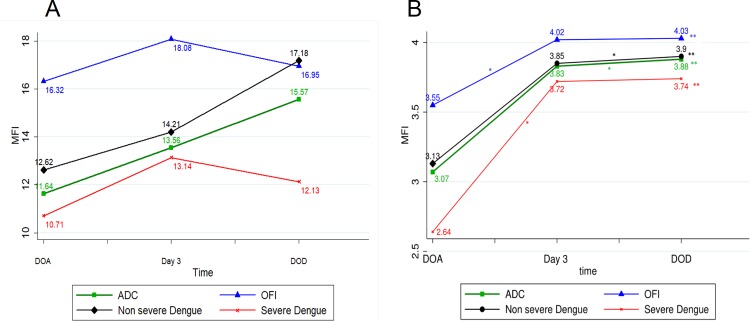
**Line graph among different groups (A) DC-SIGN (CD209) (B) FCϒR2A (CD32).** (ADC- All Dengue cases, OFI–Other febrile illness, DOA- Day of Admission, DOD- Day of Discharge) *comparison between two time points, indicates p value < 0.05 **Comparison across all three time points, indicates p value < 0.05.

In other febrile illness patients, DC-SIGN expression increased from DOA to Day 3 and then decreased from Day 3 to DOD. On contrary, FCϒR2A expression increased from DOA to Day 3 and to DOD. A statistically significant difference in FCϒR2A expression (p = 0.0001) was seen in comparison across all groups. (Fig 2A and 2B)

### DC-SIGN and FCϒR2A expression on platelets in severe dengue cases when compared with non severe

To further evaluate the role of DC-SIGN and FCϒR2A with respect to platelet involvement in dengue pathophysiology, we compared the expression of the receptors in severe and non severe dengue cases. We found that both DC-SIGN and FCϒR2A were decreased in severe dengue when compared with non severe dengue on Day of admission. Along the course of the disease, we found that DC-SIGN expression in Severe Dengue increased from DOA to Day 3 and then decreased from Day 3 to DOD. On contrary, FCϒR2A expression increased from DOA to Day 3 and to DOD. (Fig 2A and 2B) In non severe dengue patients, DC-SIGN and FCϒR2A expression increased from DOA to Day 3 and to DOD in Dengue cases.

The FCϒR2A expression on, comparison across all three time points, was found to be statistically significant both in severe dengue (p = 0.0259) and non severe dengue (p = 0.0006).

### DC-SIGN and FCϒR2A expression on platelets in patients in active viremia state

To elucidate more clearly the pattern of receptor expression on platelet surface and the viremia state of the patient, we further compared the expression of the receptors DC-SIGN and FCϒR2A on platelets based on the NS1 and IgM status of the patient. We found that both DC-SIGN and FCϒR2A were decreased in NS1 positive cases when compared with NS1 Negative cases on Day of admission.

**Fig 3:**
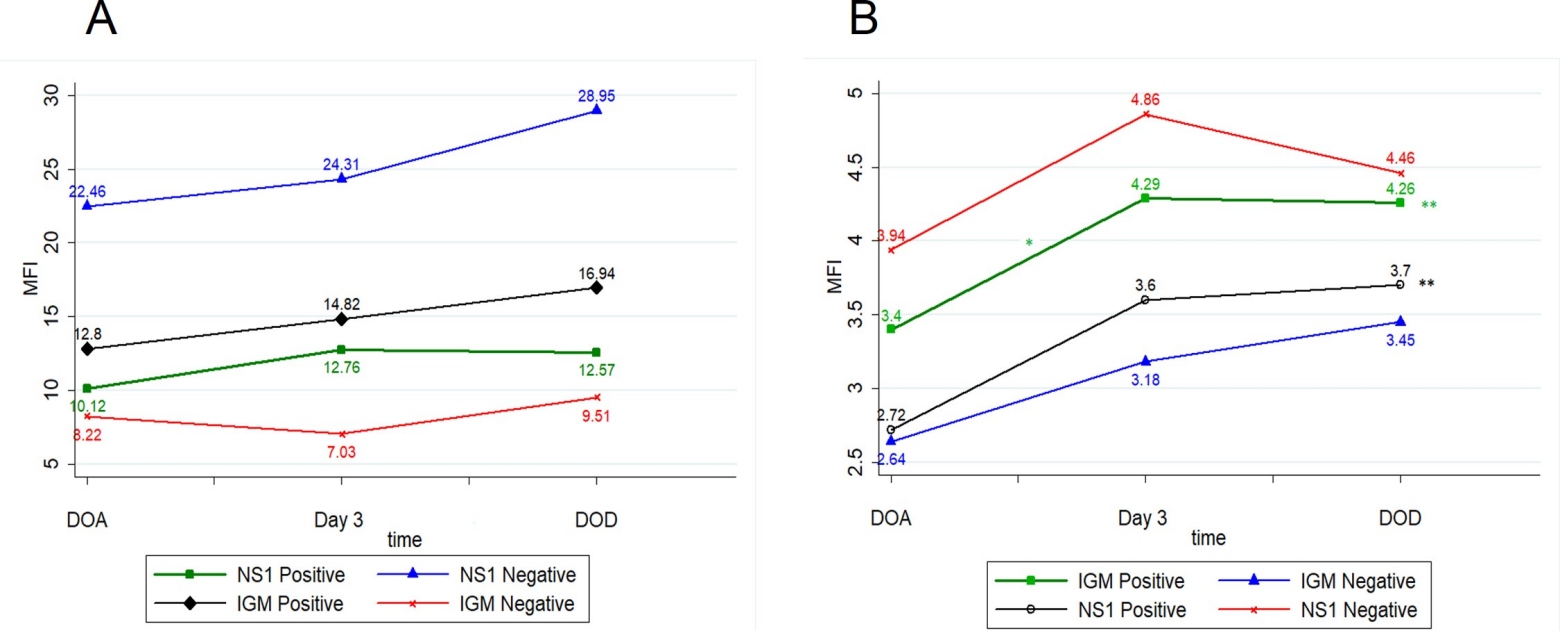
**Line graph among NS1 and IGM groups (A) DC-SIGN (CD209) (B) FCϒR2A (CD32).** (DOA- Day of Admission, DOD- Day of Discharge) *Comparison between two time points, indicates p value < 0.05 **Comparison across all three time points, indicates p value < 0.05.

In NS1 positive cases, DC-SIGN expression increased from DOA to Day 3 and then decreased from Day 3 to DOD, whereas FCϒR2A expression increased from DOA to Day 3 and to DOD. A statistically significant difference was seen in the FCϒR2A expression (p = 0.0005) on comparison across all groups. (Fig 3A and 3B)

In NS1 Negative cases, DC-SIGN expression increased from DOA to Day 3 and to DOD. FCϒR2A expression increased from DOA to Day 3 and then decreased from Day 3 to DOD.

In IgM positive cases, DC-SIGN expression increased from DOA to Day 3 and to DOD. FCϒR2A expression increased from DOA to Day 3 and then decreased from Day 3 to DOD. A statistically significant difference was seen in the FCϒR2A expression (p = 0.0001) on comparison across all groups.

In IGM negative cases, DC-SIGN expression decreased from DOA to Day 3 and then increased from Day 3 to DOD. FCϒR2A expression increased from DOA to Day 3 and to DOD. (Fig 3A and 3B)

### DC-SIGN and FCϒR2A expression on platelets in DENV RNA positive patients when compared with DENV RNA Negative patients

To explore more about the role of DENV RNA associated with platelets in the regulation of receptor expression on platelets, we undertook PCR for DENV in platelets. We proceeded to compare the data with DC-SIGN and FCϒR2A expression. Platelets were separated from the Forty-four dengue patients and RNA extraction was done. Adequate RNA (1000ng) was able to be extracted from 30 patients. When subjected to PCR, 16 samples were found to be positive for Dengue Viral RNA and were grouped as Dengue viral Positive group.

We found that both DC-SIGN and FCϒR2A receptor expression on surface of platelets was decreased in Dengue viral Positive group when compared with Dengue viral Negative group on Day of admission.

**Fig 4:**
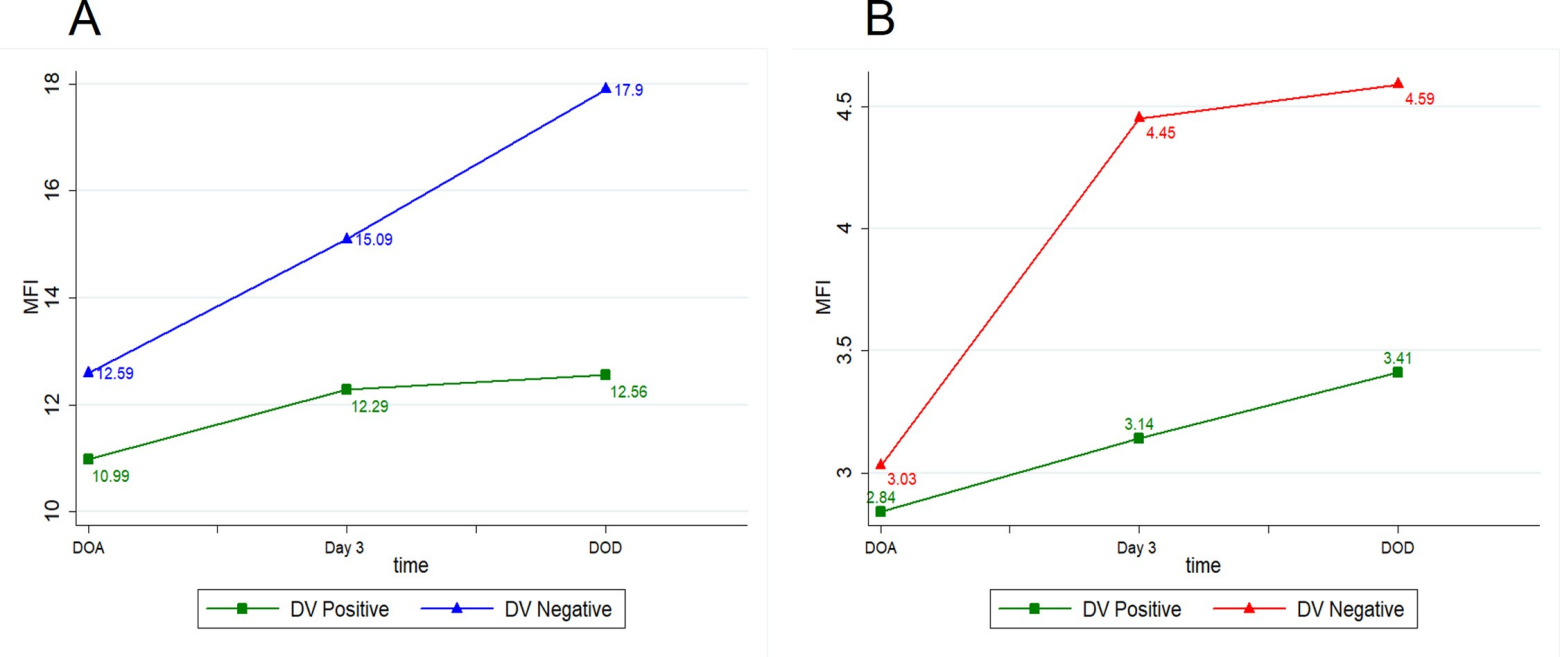
**Line graph among different DV groups (A) DC-SIGN (CD209) (B) FCϒR2A (CD32).** (DOA- Day of Admission, DOD- Day of Discharge).

DC-SIGN and FCϒR2A expression increased from DOA to Day 3 and to DOD in Dengue viral Positive group. In Dengue viral Negative group, DC-SIGN and FCϒR2A expression increased from DOA to Day 3 and to DOD. But, no statistically significant difference was observed in any of the receptor expression on comparison across all groups. (Fig 4A and 4B).

## Discussion

Platelet proteins were found to be altered in dengue. [2] Assessment of the change (if any) in platelet receptors during the course of dengue viral infection may provide new insights into dengue pathogenesis. In the current study, we found that Dengue patients had lower DC-SIGN expression on platelets when compared with OFI patients on day of admission. (Fig 1A) Further, mean DC-SIGN expression was lower in severe dengue when compared with non severe dengue. Interestingly, Dengue patients in active viremia state (NS1 Positive and IGM Negative) had decreased mean DC-SIGN expression when compared with NS1 negative and IgM Positive group.

Along the course of disease, we found that DC-SIGN expression showed increasing trend from DOA to DOD in dengue. On subgroup analysis, non severe dengue cases had DC-SIGN expression increasing from DOA to DOD. This was in contrast to severe dengue cases where mean DC-SIGN expression increased from DOA to Day 3 and then decreased on DOD. (Fig 3) Similarly, in NS1 positive patients, DC-SIGN expression increased from DOA to Day 3 and then decreased on DOD; whereas in NS1 negative patients, DC-SIGN expression showed increasing trend from DOA to DOD. (Fig 3A)

Studies have shown that dendritic cell lines expressing higher DC-SIGN are more prone to dengue viral infection. [6] Reciprocally, immature dendritic cells incubated with anti DC-SIGN antibodies exhibited higher resistance against dengue infection. [10]

Similar to their action on dendritic cells, anti DC-SIGN antibodies were found to profoundly decrease the binding of dengue virus to platelets.[11] Hence platelets with elevated DC-SIGN expression are a vulnerable target for attachment of dengue virus. Our study had shown that there was a decreased DC-SIGN expression in severe dengue as well as in active viremia states (NS1 Positive and IGM Negative). Further, in severe dengue and NS1 state, where active viremia persists, the re-enhancement of DC-SIGN on DOD was not seen.

The dengue virus interaction with platelets via DC-SIGN induces platelet activation and platelet apoptosis. In addition, Hottz et al (2013) demonstrated that incubation with anti DC-SIGN antibodies prevents the dengue viral-mediated platelet activation.[8] This activation of platelets is denoted by translocation of phosphatidyl serine and modulation of platelet proteome.[2] One of the proteins modulated by Dengue virus is Interleukin -1b. Dengue virus has been shown to induce synthesis of interleukin -1b from platelets.[12] Interleukin -1b is a proinflammatory cytokine, produced by monocytes when induced by Th1 helper cells. [13] Host immune system mounts a Th1-type response in the early period of dengue disease. The conversion to Th2-type is associated with increased severity of the disease.[14] Hence, Platelets also facilitates the host immune system by producing the proinflammatory cytokines on coming in contact with dengue virus. This cascade of cytokine activation is thought to play a major role in the development of severe disease.[15]

Collectively, our results suggest the downregulation of the DC-SIGN in dengue infection which prevents the disproportionate activation of host immune system via cytokine cascade preventing organ impairment. As dengue virus get successfully cleared out by host immune system, platelets allow the DC-SIGN to be expressed on the platelet membrane as seen in the progressively increasing expression along the course of disease in dengue patients. In active viremia states (NS1 Positive and IGM Negative) and in Severe dengue, where dengue virus persists in circulation, the DC-SIGN expression gets further down-regulated. This also points to the possible role of DC-SIGN as a prognostic marker of dengue disease progression.

Similar to DC-SIGN, FcƳR2A expression was also found to be decreased in Dengue when compared with OFI on day of admission. (Fig 1B) Further, FcƳR2A expression was lower in severe dengue when compared with non severe dengue. In dengue patients in active viremia state, the mean FcƳR2A expression was found to be decreased when compared with inactive state.

Along the course of disease, the mean FcƳR2A expression on platelets showed an increasing trend in dengue and OFI from DOA to DOD. Further, FcƳR2A expression showed an increasing trend in nonsevere as well as in severe dengue from DOA to DOD. In contrast to DC-SIGN expression, mean FcƳR2A expression didn't show a decrease by DOD in severe dengue. (Fig 4) In IGM negative group, the trend was found to be increasing from DOA to DOD. Paradoxically, in IGM positive patients, FcƳR2A expression increased from DOA to Day3 and then decreased on DOD. (Fig 3B)

Tomiyama Y et al (1992) had demonstrated FcƳR2A as the sole Fc receptor present on platelets.[16] Platelet FcƳR2A receptor binds with IgG complexes and facilitates its clearance from circulation by being phagocytosed. [17] In addition, an increased association between platelet-associated immunoglobulins, thrombocytopenia and severity of disease have also been demonstrated.[18] Similar to DC-SIGN, FcƳR2A receptor also have been found to play a role in platelet activation. [19,20]

In dengue infection, FcƳR2A receptors behave differently from other FcƳ receptors. FcƳR1 present on monocytes helps in efficient phagocytosis and neutralization of dengue virus. On contrary, FcƳR2A receptor facilitates the entry of DENV into an intracellular environment favorable for replication. [21]

In addition to above-mentioned roles of FcƳR2A receptors, they are also involved in antibody dependent enhancement of infection.[22] FcƳR2A receptors allow the internalization of IgG-containing immune complexes into the cell.[17] Hence, Platelets with elevated FcƳR2A expression are more prone to phagocytic/ADCC mediated destruction as well as enhanced dengue viral infection by ADE mechanism.

In the current study, we found that FcƳR2A receptor expression on platelets was decreased in severe dengue as well as in active viremia states (NS1 positive and IgM negative).Watanabe S et al have demonstrated the presence of enhancing antibodies in severe dengue which overcome the neutralizing antibodies and accelerate ADE leading to increased infection of FcƳR2A expressing platelet cells.[23] Both NS1 positive, as well as IGM negative states, are characterized by the presence of DENV in circulation. Hence in both these groups, there is increased formation of immune complexes which would bind to FcƳR2A receptor.

On collation, one may be tempted to hypothesize that the downregulation of the FcƳR2A receptor on platelet surface in dengue infection prevents the platelets from providing the virus a facilitatory environment and diminishes the targeting of platelet by activated host immune system, namely NK cells and Macrophages. As dengue virus get successfully cleared out by host immune system, FcƳR2A receptors are re-expressed on the platelet membrane as seen in increased expression in DOD. In active viremia states (NS1 Positive and IGM Negative) and in Severe dengue, where dengue virus persists in circulation, the FcƳR2A expression gets further down-regulated.

Platelets, being anucleated cells lack transcriptional apparatus. But they are able to translate host mRNA to proteins. Brown GT et al (2011) have shown that platelets do produce proteins de novo.[24] Further, splicing of mRNA have also been demonstrated in platelets. [25] These processess are accelerated in activated platelets. Various viral RNA has been shown to be present in platelets. Noisakran S et al(2009) had demonstrated the presence of Dengue viral RNA in platelets. [26] Dengue like viral particles have been shown by electron microscopy to be present in platelets. Dengue viral RNA utilizes the host cells machinery to produce infectious virions. In this regard, Simon AY et al had shown the release of NS1 proteins from platelets infected with dengue virus.[11] Further, Sutherland MR et al have shown that dengue virus can replicate in stored platelets.[27] Comparison of flow cytometry parameters with DENV RNA status in platelets can show light on the influence of viral RNA on the receptor expression on the platelet surface.

In the current study, we found that both DC-SIGN and FcƳR2A expression was decreased in DENV RNA positive patients when compared with DENV RNA negative patients. (Fig 4A and 4B) Statistically significant difference was not observed in both cases.

DENV utilizes DC-SIGN and FcƳR2A to gain entry into platelets. DENV RNA in platelets uses platelet translation machinery to produce viral proteins. These viral proteins help in formation of infective virions which subsequently infect other cells.[28] These viral proteins also accelerate the activation of host immune system. Higher NS1 in circulation is related to increased severity of disease.[29] Simon AY et al (2015) have postulated that the retention of these viral proteins makes platelets target for immune mediated destruction.[11] Studies also demonstrates DENV RNA in platelets leading to increased monocyte-platelet complex leading to platelet clearance.[30] Further, high DENV genome copies have been shown to lead to platelet activation and increased complement deposition. [31]

## Conclusion

Collectively, It may be concluded that the two receptors, DC-SIGN and FcƳR2A, which are utilized by Dengue virus to gain entry into the cell, had their expression on platelet surface suppressed in patients with higher titre of circulating dengue virus. Derepressions of surface expression of the mentioned receptors were evident with the clearance of dengue virus from circulation. It also points to the future possible role of DC-SIGN as a prognostic indicator of dengue disease progression. The decreased expression of the receptors DC-SIGN and FcƳR2A on platelet surface allow the platelet to evade the activated host immune system as well as deny safe haven for dengue virus from immune mediated clearance.

## Limitations

Various strains of DENV were not studied separately though different serotypes of DENV have different virulence and hence interact uniquely with CD209 and CD32 on platelets. The types of intervention/ management/ medication were not taken into consideration among study subjects which may act as a confounding factor. Number of patients recruited in pediatric age group was limited. We were not able to assess the gene expression of the receptors or serum levels of the same for further confirmation.

### Scope for future study

Assessment of the genetic regulation of receptor expression on platelet would reveal the exact nature of downregulation of receptor on platelet surface. Also, the serum levels of same receptor can points to the shedding of the receptor from platelet surface. Further studies are also required to find out the mechanism by which the changes in receptor expression are brought forth.

## Supporting information

S1 File Supplementary data. (DOCX)
