# Supplementary material for: Dynamic modulation of DC-SIGN and FcΥR2A receptors expression on platelets in dengue
Source: PLoS One. 2018 Nov 9;13(11):e0206346. doi: 10.1371/journal.pone.0206346 (PMC6226166; doi:10.1371/journal.pone.0206346)
Supplement: S1 File — (DOCX) [file pone.0206346.s001.docx]

**Supplementary Data**

**S1 Table. Comparison of MFI of CD 209 and CD32 across all groups on day of admission**

| Flow Cytometry  Parameters | All dengue  N=44 | Other Febrile illness N= 44 | Non febrile illness  N=15 |
| --- | --- | --- | --- |
| CD209 (MFI) | 11.64  (7.64-20.13) | 16.32  (10.29-25.97) | 20.75  (18.02-24.67) |
| p value | 0.026* | | |
| CD32(MFI) | 3.07  (2.37-4.6) | 3.55  (2.66-4.46) | 4.12  (3.9-4.2) |
| p value | 0.141 | | |

*p value <0.05 – statistical significance

Statistical Test :Kruskal Wallis Rank test with Dunn’s post-test

**S2 Table. Comparison of MFI of CD209 and CD32 on Platelets in Dengue patients along the course of time .**

| Flow Cytometry  Parameters | All dengue  N=44 | | |
| --- | --- | --- | --- |
|  | DOA | DAY 3 | DOD |
| CD209(MFI) | 11.64  (7.64-20.13) | 13.56  (10.19-22.28) | 15.57  (9.99-24.17) |
| p value | 0.178 | | |
| CD32(MFI) | 3.07  (2.37-4.6) | 3.83  (2.9-5.29) | 3.88  (3.23-5.48) |
| p value | 0.0001* | | |

(DOA- Day of Admission , DOD- Day of Discharge)

Statistical Test : Friedman test with Dunn’s post-test

*p value <0.05 – statistical significance

**S3 Table. Comparison of MFI of CD209 and CD32 on Platelets in Other febrile illness group along the course of time .**

| Flow Cytometry  Parameters | Other Febrile illness N= 44 | | |
| --- | --- | --- | --- |
|  | DOA | DAY 3 | DOD |
| CD209(MFI) | 16.32  (10.29-25.97) | 18.08  (12.41-24.62) | 16.95  (10.56-26.63) |
| p value | 0.6205 | | |
| CD32(MFI) | 3.55  (2.66-4.46) | 4.02  (3.18-5.36) | 4.02  (3.18-5.36) |
| p value | 0.0001* | | |

(DOA- Day of Admission , DOD- Day of Discharge)

Statistical Test : Friedman test with Dunn’s post-test

*p value <0.05 – statistical significance

**S4 Table. Comparison of MFI of CD209 and CD32 on Platelets in Severe Dengue patients along the course of time.**

| Flow Cytometry  Parameters | Severe dengue  N= 10 | | |
| --- | --- | --- | --- |
|  | DOA | DAY 3 | DOD |
| CD209(MFI) | 10.71  (7.56-13.28) | 13.14  (12.45-16.44) | 12.13  (9.51-15.91) |
| p value | 0.8302 | | |
| CD32(MFI) | 2.64  (2.37-4.14) | 3.72  (3.14-4.74) | 3.74  (3.08-5.21) |
| p value | 0.0259* | | |

(DOA- Day of Admission , DOD- Day of Discharge)

Statistical Test : Friedman test with Dunn’s post-test

*p value <0.05 – statistical significance

**S5 Table.Comparison of MFI of CD209 and CD32 on Platelets in Non Severe Dengue patients along the course of time.**

| Flow Cytometry  Parameters | Non severe dengue  N=34 | | |
| --- | --- | --- | --- |
|  | DOA | DAY 3 | DOD |
| CD209(MFI) | 12.62  (8.22-20.49) | 14.21  (10.03-23.31) | 17.18  (10.39-28.42) |
| p value | 0.1394 | | |
| CD32(MFI) | 3.13  (2.38-4.62) | 3.85  (2.7-5.44) | 3.95  (3.31-5.64) |
| p value | 0.0006* | | |

(DOA- Day of Admission , DOD- Day of Discharge)

Statistical Test : Friedman test with Dunn’s post-test

*p value <0.05 – statistical significance

**S6 Table. MFI of CD209 and CD32 on Platelets in NS1 Positive Dengue patients along the course of time.**

| Flow Cytometry | NS1 Positive  N=29 | | |
| --- | --- | --- | --- |
|  | DOA | DAY 3 | DOD |
| CD209(MFI) | 10.12  (7.09-17.19) | 12.76  (9.07-17.88) | 12.57  (9.39-20.13) |
| p value | 0.1220 | | |
| CD32(MFI) | 2.72  (2.37-4.14) | 3.60  (2.98-4.41) | 3.7  (3.08-4.92) |
| p value | 0.0005* | | |

(DOA- Day of Admission, DOD- Day of Discharge)

Statistical Test : Friedman test with Dunn’s post-test

*p value <0.05 – statistical significance

**S7 Table. MFI of CD209 and CD32 on Platelets in NS1 Negative Dengue patients along the course of time.**

| Flow Cytometry  Parameters | NS1 Negative  N=10 | | |
| --- | --- | --- | --- |
|  | DOA | DAY 3 | DOD |
| CD209(MFI) | 22.46  (9.76-80.58) | 24.81  (15.77-63.97) | 28.95  (13.18-57.79) |
| p value | 0.8302 | | |
| CD32(MFI) | 3.94  (2.26-6.74) | 4.86  (2.71-6.54) | 4.46  (3.38-6.25) |
| p value | 0.4362 | | |

(DOA- Day of Admission, DOD- Day of Discharge)

Statistical Test : Friedman test with Dunn’s post-test

p value <0.05 – statistical significance

**S8 Table. MFI of CD209 and CD32 on Platelets in IGM Positive Dengue patients along the course of time.**

| Flow Cytometry  Parameters | IGM Positive  N=32 | | |
| --- | --- | --- | --- |
|  | DOA | DAY 3 | DOD |
| CD209(MFI) | 12.84  (8.02-20.89) | 14.82  (11.61-22.28) | 16.94  (12.13-25.30) |
| p value | 0.5523 | | |
| CD32(MFI) | 3.4  (2.23-4.65) | 4.29  (2.91-5.99) | 4.26  (3.31-5.92) |
| p value | 0.0001* | | |

(DOA- Day of Admission, DOD- Day of Discharge)

Statistical Test : Friedman test with Dunn’s post-test

*p value <0.05 – statistical significance

**S9 Table. MFI of CD209 and CD32 on Platelets in IGM Negative Dengue patients along the course of time.**

| Flow Cytometry  Parameters | IGM Negative  N=5 | | |
| --- | --- | --- | --- |
|  | DOA | DAY 3 | DOD |
| CD209(MFI) | 8.22  (7.56-15.68) | 7.03  (6.5-23.25) | 9.51  (8.97-20.13) |
| p value | 0.0934 | | |
| CD32(MFI) | 2.64  (2.57-2.91) | 3.18  (2.62-3.28) | 3.45  (3.08-3.60) |
| p value | 0.3311 | | |

(DOA- Day of Admission, DOD- Day of Discharge)

Statistical Test : Friedman test with Dunn’s post-test

p value <0.05 – statistical significance

**S10 Table. Dengue Viral RNA detected from platelets- Summary**

| Dengue Viral RNA | N | Percentage |
| --- | --- | --- |
| Present | 16 | 53.33 |
| Absent | 14 | 46.67 |
| Total | 30 | 100 |

**S11 Table. Comparison of Dengue Viral RNA with NS1 status among Dengue patients:**

| **Dengue Viral RNA** | NS1 Present (N) | NS1Absent (N) | p value |
| --- | --- | --- | --- |
| **Present** | 10 | 4 | 0.410 |
| **Absent** | 11 | 2 |  |

**Comparison of Dengue Viral RNA with IgM status among Dengue patients:**

| **Dengue Viral RNA** | IgM Present (N) | IgM Absent (N) | p value |
| --- | --- | --- | --- |
| **Present** | 10 | 3 | 0.089 |
| **Absent** | 11 | 0 |  |

Statistical Test : Pearson’s Chi Square test

**S12 Table. Comparison of Dengue Viral RNA with Severity of Dengue among Dengue patients:**

| **Dengue Viral RNA** | Non Severe (N) | Severe (N) | p value |
| --- | --- | --- | --- |
| **Present** | 11 | 5 | 0.544 |
| **Absent** | 11 | 3 |  |

Statistical Test : Pearson’s Chi Square test

**S13 Table. MFI of CD209 and CD32 on Platelets in Dengue viral positive group along the course of time.**

| Flow Cytometry  Parameters | DV positive  N=16 | | |
| --- | --- | --- | --- |
|  | DOA | DAY 3 | DOD |
| CD209(MFI) | 10.99  (7.64-17.96) | 12.29  (8.43-16.85) | 12.56  (9.24-17.64) |
| p value | 0.8290 | | |
| CD32(MFI) | 2.84  (2.44-4.38) | 3.14  (2.66-4.25) | 3.41  (3.014.24) |
| p value | 0.0681 | | |

(DOA- Day of Admission, DOD- Day of Discharge)

Statistical Test : Friedman test with Dunn’s post-test

p value <0.05 – statistical significance

**S14 Table. MFI of CD209 and CD32 on Platelets in Dengue viral negative group along the course of time.**

| Flow Cytometry  Parameters | DV negative  N=14 | | |
| --- | --- | --- | --- |
|  | DOA | DAY 3 | DOD |
| CD209(MFI) | 12.59  (9.76-27.97) | 15.09  (12.82-23.31) | 17.9  (10.45-24.2) |
| p value | 0.7515 | | |
| CD32(MFI) | 3.03  (2.37-5.87) | 4.45  (3.5-5.93) | 4.59  (2.9-5.97) |
| p value | 0.0617 | | |

(DOA- Day of Admission, DOD- Day of Discharge)

Statistical Test : Friedman test with Dunn’s post-test

p value <0.05 – statistical significance

**S15 Table. Comparison of CD209 in dengue subgroups**

| **Groups** | **CD 209** | | | **p Value** |
| --- | --- | --- | --- | --- |
| **Severity of Dengue** |  | **Non Severe** | **Severe** |  |
|  | **DOA** | 12.62  (8.22-20.49) | 10.71  (7.56-13.28) | 0.385 |
|  | **DAY 3** | 14.21  (10.03-23.31) | 13.14  (12.45-16.44) | 0.604 |
|  | **DOD** | 17.18  (10.39-28.42) | 12.13  (9.51-15.91) | 0.104 |
| **NS1** |  | **NS1 Positive** | **NS1 Negative** |  |
|  | **DOA** | 10.12  (7.09-17.19) | 22.46  (9.76-80.58) | 0.057 |
|  | **DAY 3** | 12.76  (9.07-17.88) | 24.81  (15.77-63.97) | 0.007* |
|  | **DOD** | 12.57  (9.39-20.13) | 28.95  (13.18-57.79) | 0.012* |
| **IgM** |  | **IgM Positive** | **IgM Negative** |  |
|  | **DOA** | 12.84  (8.02-20.89) | 8.22  (7.56-15.68) | 0.563 |
|  | **DAY 3** | 14.82  (11.61-22.28) | 7.03  (6.5-23.25) | 0.266 |
|  | **DOD** | 16.94  (12.13-25.30) | 9.51  (8.97-20.13) | 0.230 |
| **Dengue Viral RNA** |  | **Positive** | **Negative** |  |
|  | **DOA** | 10.99  (7.64-17.96) | 12.59  (9.76-27.97) | 0.677 |
|  | **DAY 3** | 12.29  (8.43-16.85) | 15.09  (12.82-23.31) | 0.124 |
|  | **DOD** | 12.56  (9.24-17.64) | 17.9  (10.45-24.2) | 0.244 |

Statistical Test : Wilcoxon rank sum ( Mann Whitney ) test

*p value <0.05 – statistical significance

**S16 Table.Comparison of CD32 in dengue subgroups**

| **Groups** | **CD 32** | | | **p Value** |
| --- | --- | --- | --- | --- |
| **Severity of Dengue** |  | **Non Severe** | **Severe** |  |
|  | **DOA** | 3.13  (2.38-4.62) | 2.64  (2.37-4.14) | 0.385 |
|  | **DAY 3** | 3.85  (2.7-5.44) | 3.72  (3.14-4.74) | 0.705 |
|  | **DOD** | 3.95  (3.31-5.64) | 3.74  (3.08-5.21) | 0.556 |
| **NS1** |  | **NS1 Positive** | **NS1 Negative** |  |
|  | **DOA** | 2.72  (2.37-4.14) | 3.94  (2.26-6.74) | 0.281 |
|  | **DAY 3** | 3.60  (2.98-4.41) | 4.86  (2.71-6.54) | 0.203 |
|  | **DOD** | 3.7  (3.08-4.92) | 4.46  (3.38-6.25) | 0.288 |
| **IgM** |  | **IgM Positive** | **IgM Negative** |  |
|  | **DOA** | 3.4  (2.23-4.65) | 2.64  (2.57-2.91) | 0.578 |
|  | **DAY 3** | 4.29  (2.91-5.99) | 3.18  (2.62-3.28) | 0.045* |
|  | **DOD** | 4.26  (3.31-5.92) | 3.45  (3.08-3.60) | 0.083 |
| **Dengue Viral RNA** |  | **Positive** | **Negative** |  |
|  | **DOA** | 2.84  (2.44-4.38) | 3.03  (2.37-5.87) | 0.632 |
|  | **DAY 3** | 3.14  (2.66-4.25) | 4.45  (3.5-5.93) | 0.073 |
|  | **DOD** | 3.41  (3.01-4.24) | 4.59  (2.90-5.97) | 0.270 |

Statistical Test : Wilcoxon rank sum ( Mann Whitney ) test

*p value <0.05 – statistical significance
